# Exploring Implicit and Explicit Attitudes of Employees’ Authentic Organizational Loyalty

**DOI:** 10.3389/fpsyg.2021.666869

**Published:** 2021-05-19

**Authors:** Ji-Woong Hong, Ah Jeong Hong, Sang Rak Kim

**Affiliations:** ^1^Department of Human Resource Development, Chung-Ang University, Seoul, South Korea; ^2^Department of Education, Chung-Ang University, Seoul, South Korea

**Keywords:** organizational loyalty, Implicit Association Test, implicit attitude, explicit attitude, South Korea

## Abstract

The purpose of this study was to develop the OL-IAT (Organizational Loyalty – Implicit Association Test), an IAT tool that measures implicit attitudes toward organizational loyalty, and to verify its predictive validity. In total, 15 respondents were randomly selected from among the employees of P Company. The respondents were then asked open questions about the image they held of the target company. Based on their responses, a list of words was compiled for use on the OL-IAT. Evaluation categories, either like-dislike or positive-negative, were developed for this test based on the approach-avoidance words proposed by Ostafin and Palfai (2006). The OL-IAT was then administered to 127 employees of Company P, and self-reporting measures were also devised to measure explicit attitudes. A survey of whether the implicit attitude toward organizational loyalty was consistent with the explicit attitude showed that there was no significant correlation, and the attitude inconsistency was evident in the responses. When differences in rank were accounted for, organizational loyalty attitudes were consistent at the manager level, and organizational participation attitudes were consistent at the manager and general staff levels. This study will aid in the development of effective measures of organizational loyalty and accelerate interventions to increase it, which in turn will positively impact long-term organizational growth.

## Introduction

Retaining talented employees is considered a driving force for organizational success, and, for this reason, people commonly refer to the ongoing “talent war” between companies. To win the talent war, companies must make every effort to retain employees and turnover rates. In the United States, workers have a short average tenure of about 4.2 years, and in the United Kingdom, average tenure tends to be very short throughout an individual’s career. Despite the common notion of “lifetime work” in Asian countries, similar trends prevail. In 2017, 311 million people moved to Japan, a figure which had steadily increased for 7 years in a row; even in Korea, despite very high levels of youth unemployment, the retirement rate of new employees with fewer than 4 years of service is more than 50%. This results in huge losses for employers (Statistics Korea(KOSTAT), 2019; Wall Street Journal, 2018), which will ultimately affect the economy as a whole. In light of these statistics, increasing employees’ organizational loyalty has increasingly become the focus of research efforts, and is recognized as a crucial factor for effective talent management and organizational development.

Organizational loyalty can be defined as a sincere dedication to an organization. It engenders a sense of identity and self-sacrifice at the same time, engaging an employee mentally and physically and also requiring a commitment of their time to the organization. It is an attitude that compels the employee to act in accordance with the norms and values of the organization (Ladd, 1967; Ashforth and Mael, 1989). Blau (1986) found that loyalty to an organization correlates positively with low turnover rates and improved ability to achieve goals, and Caldwell et al. (1990) also found that loyalty to an organization corresponds with strong personal identification with that organization. Positive effects of building a strong organizational culture occur both individually and systematically. Bailyn (1993) argued that strong trust in an organization motivates employees to perform their duties and instills a sense of responsibility to the organization, thereby increasing organizational loyalty. In many business administration and HRD (human resource development) studies, organizational loyalty naturally emerges as an important factor in the corporate scene; indeed, Baldwin et al. (2001) argued that it is an essential factor for employee performance and commitment.

This leaves us with a fundamental question: are there other ways to identify employees who are the most likely to be loyal to their companies? The majority of tests that measure organizational loyalty rely on self-reported questionnaires that are inherently subjective and cannot completely capture the true nature of employees’ feelings. When filling out organizational loyalty questionnaires, employees may worry that if their loyalty scores are low, it will place them at a disadvantage or open them to negative evaluations within the organization, and their answers may be influenced by these anxieties or doubts. In previous studies, the majority of people tended to consciously distort their responses in order to appear more morally sensitive to uncomfortable issues such as race, gender, and identity that demand “social desirability” (Greenwald and Banaji, 1995; Greenwald et al., 1998; Tam et al., 2010; Han et al., 2017). Therefore, testing organizational loyalty should be done in a way that limits opportunities for conscious judgment and promotes automatic and sincere responses. For this, it is necessary to measure unconscious impressions, not consciously censored ones. The “Implicit Association Test (IAT)” is one such way to assess these unconscious opinions, as it is often possible to predict deep thoughts, intentions, and behaviors by examining general human attitudes.

Attitudes can be divided into explicit attitudes and implicit attitudes. An explicit attitude is a response based on conscious judgment and can be measured directly by self-report. On the other hand, implicit attitudes are unconscious biases that cannot be consciously perceived and are therefore investigated using cognitive measures or experimental methods such as the IAT and the Dot probe (Wojnowicz et al., 2009). Since an explicit attitude is a conscious reaction, false or distorted responses are possible. On the other hand, falsely reporting an implicit attitude is more difficult to do (Fazio et al., 1995). Therefore, assessing implicit attitudes using these cognitive measures can provide richer information on employee attitudes because the questions that appear on these assessments are designed to reflect a broader range of essential human attitudes than responses to a simple survey questionnaire.

The purpose of the IAT is to measure implicit attitude. Implicit attitude includes one’s unconscious thoughts, intentions, dispositions, and responsiveness. However, explicit attitude is a reaction originating in conscious thinking, and it is measured by a self-report questionnaire. In prior IAT studies, it is common to examine the correlation with self-report questionnaires. Validation is also progressed through correlation, and meaning for responses is also derived. In other words, the implicit attitude implies one’s unconscious organizational loyalty, organizational commitment, and intention to turnover. Therefore, the study investigated the correlation with the self-reported questionnaire, which contains one’s conscious and explicit attitude. Among the self-report items (scale) that measure one’s explicit attitudes, we also examine the correlation with scales that have a close meaning or attribute.

IAT studies (i.e., McConnell and Leibold, 2001; Hofmann et al., 2005; Yovel and Friedman, 2013; Harrison and Lakin, 2018) interpreted the correlation and association between one’s implicit attitude evaluated by IAT and explicit attitude evaluated by self-reported questionnaire. In these studies, questions in a self-reported questionnaire were not named as explicit questions. Those questions were considered as the questions that contain variables of one’s attitude. Examining the correlation of similar self-report questionnaires to IAT outcomes and attitudes is the most widely practiced method in IAT studies.

Studies of the efficacy of the IAT have, as expected, revealed that implicit attitude (the unconscious attitude) is often at odds with the consciously revealed attitude. When applied to attitudes about sensitive subjects, Gilbert and Ricketts (2008) found that conscious responses and IAT results were quite different. Many similar studies have also revealed implicit racial prejudice. For example, in a study by Wojnowicz et al., photographs of either Black or white people were presented to participants on a computer screen where categories indicating positive or negative evaluations were present. They were simultaneously presented with either positive or negative words and were asked to sort them, along with the photographs, to either preference category. When explicit attitudes as to how the questionnaire should be answered were measured, the race of the subjects of the photographs did not result in a significant difference. However, on the IAT, the participants took more time selecting positive words when presented with photographs whose subjects were Black than when the subjects of the photos were white (Wojnowicz et al., 2009). In a study examining perceptions of the elderly, the implicit attitudes were consistent, while explicit attitudes were inconsistent (Vaes et al., 2003; Tam et al., 2010; Lee and Bang, 2012). Therefore, investigating an implicit attitude is more likely to yield a consistent and true response. Since the explicit attitude corresponding to a conscious reaction and the implicit attitude representing an unconscious reaction can differ, the IAT must necessarily be used to investigate implicit attitudes that exclude conscious judgment, as it permits us to obtain more genuine responses. In addition to being less prone to conscious manipulation by respondents, IAT scores are easy to quantify and can be standardized using statistical analysis. The IAT has been used with continually increasing frequency as its administration has been made easier by networks like the Internet (Cho and Bang, 2003; Han et al., 2017).

Therefore, in order to develop and verify an Organizational Loyalty test using the IAT, the research outlined here proceeded in three phases. First, an IAT test tool, OL-IAT (Organizational Loyalty-IAT), was developed to measure implicit attitudes toward organizational loyalty. Second, the correlation between implicit and explicit attitudes toward organizational loyalty was evaluated. Finally, scores were used to evaluate the consistency of attitudes according to an employee’s position in the organization.

## Research Method

### Participants

The study was conducted on employees who work for “P company” in Korea, which is one of the largest steel manufacturing companies in the world. For the development of OL-IAT, structured questions were answered by 15 employees from P company and 3 employees from other companies. The OL-IAT developed in this way was then administered to 127 employees (109 males and 18 females) of “P company” who attended an in-house training course. Employees’ ranks (classes) were of three types: staff, assistant manager, or manager. The average age was 30.23 years old (SD = 7.06). Reflecting the wider demographics of the steel industry, the proportion of male employees was high. Employees participated in this study while completing a training program. After the initial training session, participants took breaks of at least 30 min prior to completing experimental tests. Office supplies and refreshments were provided as compensation for participation. In order to observe research ethics, we conducted these experiments in accordance with ethical procedures implemented in prior studies.

### Procedure

First, appropriate words tailored specifically to the development of the OL-IAT were selected. Target categories symbolizing the company were randomly selected based on the responses of 15 employees of the “P company” to open-ended questions about the images and thoughts they associated with the company. In addition, researchers obtained similar responses from three employees of “S company” as a source of comparison to non-corporate employees. “S company” is a representative global company in Korea, and attracts a high volume of prospective employees as it is regarded as a desirable place to work. Following are the rationale for selecting S Company as a comparison target for P Company. First, both P and S companies have achieved USD 9 billion (KRW 10 trillion) in sales and USD 900 million (KRW 1 trillion) in operating profits over the last 20 years in Korea. Also, both companies have similar management scales that can be compared to each other. Second, both companies played a key role in Korea’s rapid economic growth in the 1960s and 1970s in that Korean people regard common factors in business image and situation and tend to compare them often. Third, in terms of thoughts and attitudes of employees, both companies require similar conditions and periods for their recruitment. According to many employment agencies, college students have chosen two companies as the most wanted company to join. Fourth, company P employees often compare their company with S when determining the level of compensation for their work. It is because both companies have similar wages, welfare policies, and working conditions. In order to verify comparison selection, 50 employees of company P were asked, and 42 of them (84%) indicated company S.

Evaluative categories were selected in the development of the test to capture like-dislike or positive-negative associations, based on the approach-avoidance words proposed by Ostafin and Palfai (2006) for the development of such tests. On the list of approach-avoidance words, an approach word indicates an association that is preferred and desired, and an avoidance word indicates an association that is avoided and desired. Approach-avoidance words can be a powerful way to predict real behavior because they strongly embody motivational traits that can trigger behavior or that characterize addiction. Once the OL-IAT had been developed, it was administered to 127 employees of “P company,” and a self-report scale was administered simultaneously to measure explicit attitudes. In this way, we measured both the implicit and explicit attitudes of employees toward “P company.” Researchers examined the correlation between OL-IAT and self-reported tests using statistical analysis of the responses and confirmed the degree of agreement among subjects’ responses by rank (class).

### IAT

The IAT (Implicit Association Test) is a method of interpreting the implicit attitudes that represent unconscious intentions by defining the strength of association between words as a function of response time (Wojnowicz et al., 2009). The basic premise of the test is that word stimuli are presented on a computer monitor and participants respond. There are two types of words that are used as word stimuli: “target categories” and “evaluative categories.” Target categories include words that symbolize or represent the objects being experimentally assessed. For example, if we want to characterize “United States,” “stars and stripes,” “the White House,” or “the Statue of Liberty” might all appear in the target category. Evaluative categories are comprised of pairs of words that can indicate preferences for target categories. Examples of evaluative word pairs include good-dislike, preference-hate, and approach-avoidance. When combinations of these target category words and evaluative category word pairs are presented together, the intensity of the association between them is inferred based on the time taken by the participant to respond to the words, with the respondent’s implicit attitude corresponding to the physical trajectory of their hand movements when assigning the targets to either of the words in the evaluative pair. This is based on the concept of psychological analogy, which holds that if a person’s psychological incentive is strong, it strengthens the association they form between two stimuli and shortens their reaction time. Lower incentive, conversely, leads to a weaker association and a slower response (Greenwald and Banaji, 1995; Greenwald et al., 1998). There are two types of IAT: the standard “IAT,” as we have described here, and the “SC-IAT (Single Category IAT).” SC-IAT examines the attitudes of a single subject. The general IAT can reveal relative preferences between target categories, but the SC-IAT can detect the absolute preference for one target. Both approaches are widely used in the literature (Karpinski and Steinman, 2006).

The IAT (Standard version) evaluates relative preference and approach. It measures the respondent’s opinion of which objects or concepts are more favorable or preferred relative to other items on the assessment. In the present study, a renowned Korean company (S company), which is often discussed within the target company (P company), was set as a standard for comparison. When scoring IAT responses, attitudes and thoughts that were consistent with what was overtly indicated by employees were judged in comparison to the items that appeared for comparison on the assessment.

SC-IAT (Single category version) evaluates one’s absolute preference and approach. It measures individual familiarity and preference as an independent value, without comparison to others’ responses. A significant positive correlation between SC-IAT responses and outward attitudes indicates that the participant’s absolute attitude and stated thoughts are consistent. SC-IAT responses indicated that the participants in this study have a positive impression of the steel company they belong to, based on the strength of the correlations we observed.

## Assessment Tools

### OL-IAT (Organizational Loyalty-Implicit Association Test)

**FIGURE 1:**
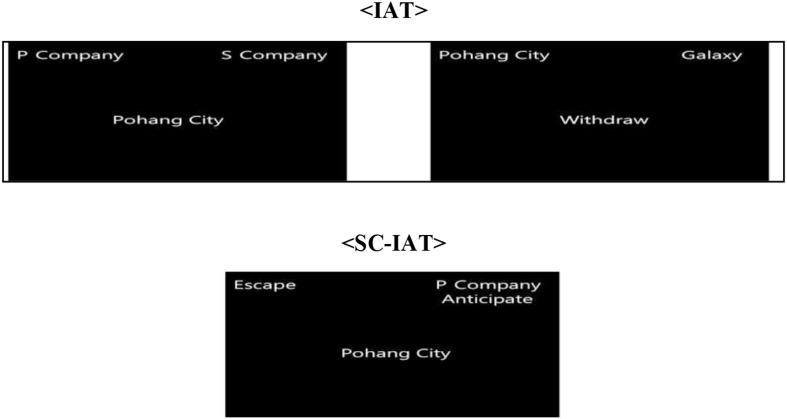
Example of IAT and SC-IAT.

Researchers have developed the OL-IAT as a new tool for determining organizational loyalty by measuring implicit attitudes corresponding to unconscious biases. Words selected for the test are listed in Table 1, and the order of word presentation is outlined in Tables 2-1, 2-2. A total of 15 respondents were randomly selected from among the employees of company P. The respondents were then asked open questions about the image they held of the target company. Their responses are stimulation words. A representative image of the screen during testing is shown in Figure 1. Each step is referred to as a “block,” and each block has different rules. Feedback is based on the participant’s correct answer. In the case of an incorrect answer, “X” is displayed on the screen and the response time for that answer is recorded as the average value of the block plus 400 ms. All reactions are constrained to a time limit of 350 to 1,500 ms, and the first and second trials of each block were usually excluded from the analysis because the reaction time was slower as the participant acclimated to the task. The ratio of stimulus presentation was adjusted to balance the number of left and right keyboard responses. These parameters were set in accordance with established standards of IAT research methods. The test-retest reliability was 0.84 for the IAT and 0.81 for the SC-IAT.

**TABLE 1:** Stimulation words.

Target categories		Evaluative categories	
Company P	Company S	Approach	Avoidance
Steel boguk	Galaxy	Advance	Away
Corporate citizen	Semiconductor	Hope	Withdraw
Chairman, Park	Anycall	Anticipate	Avoid
Pohang City	Seocho Building	Approach	Leave
Hierarchy	Magic Station	Closer	Escape

**TABLE 2-1:** Procedure of IAT.

Block	Trial	Task description	Left	Right
1	24	Initial target-category discrimination	Company P	Company S
2	24	Evaluative-categories discrimination	Avoidance	Approach
3	24	Practice	Company P + Avoidance	Company S + Approach
4	40	Perform	Company P + Avoidance	Company S + Approach
5	24	Reverse Initial target-category discrimination	S company	P company
6	36	Practice	Company S + Avoidance	Company P + Approach
7	40	Perform	Company S + Avoidance	Company P + Approach

**TABLE 2-2:** Procedure of IAT.

Block	Trial	Task description	Left	Right
1	17	Practice	Company P + Approach	Avoidance
2	51	Perform	Company P + Approach	Avoidance
3	17	Practice	Approach	Company P + Avoidance
4	51	Perform	Approach	Company P + Avoidance

### Organizational Loyalty

The Organizational Loyalty Test developed by Cole (2000) measures one’s loyalty toward an organization. A total of 15 questions appear on the test. Each question is scored on 5-point Likert scale on which the strength of a participant’s agreement with a given statement is ranked from 1 (Strongly Disagree) to 5 (Strongly Agree). Higher scores indicate higher organizational loyalty of the respondent. Items include “I am willing to sacrifice myself for the benefit of the company” and “Observe rules and regulations autonomously without boss control.”

### Organizational Engagement

The Organizational Engagement Test developed by Allen and Meyer (1990) measures one’s level of engagement with an organization. The test consists of a total of 13 questions. Each question is scored using a 5-point Likert scale as described above. Higher scores indicate a higher degree of engagement in the organization. Items include “I think there is an obligation to remain in the organization regardless of the compensation.” and “I’ve been working hard on my personal and organizational development beyond what I expected from me.”

### Turnover Intention

The turnover measure, based on the work of Michaels and Spector (1982) and Mobley (1982), measures attitudes toward transition between careers or jobs. It is comprised of a total of 7 questions, each ranked on a 5-point Likert scale, and higher scores indicate a higher propensity for employee turnover. Items include “I am considering a new job” and “If you move the company, I think it is very likely.”

## Results

### Result of IAT Test: “D”

The results of the IAT are determined by calculating the “D” score, which represents the standardized response time. The resulting D scores are normalized to an average of 0 and a standard deviation of 1. The D score has also been calculated and used to interpret results in previous IAT studies. The D score is defined as the average value of the standard score of the response time difference during the “perform” block (block 4, 7) and the standard score of the response time difference of the “practice” block (block 3, 6). The D score of the SC-IAT is computed as the standardized score for the response time difference during the “perform” block (block 2, 4).

### Descriptive Statistics

Descriptive statistics for the participants are shown in Table 3-1. The total number of employees was 127 (109 male, 18 female), and the average age was 30.23 (SD = 7.06). Descriptive statistics for the scores on each of the independent measures evaluated are shown in Table 3-2.

**TABLE 3-1:** Descriptive statistics of participants.

	Total	Man	Woman	Average age	Standard deviation	Minimum	Maximum
Total (N)	127	109	18	30.23	7.06	22	48
Staff	72	59	13	24.97	1.83	22	34
Assistant Manager	19	17	2	31.21	2.62	27	38
General Manager	36	33	3	40.22	2.98	36	48
							

**TABLE 3-2:** Descriptive statistics of results.

	Average	Standard deviation	Minimum	Maximum	Range
Organizational loyalty	62.92	6.74	41	75	34
Organizational engagement	49.48	6.29	35	64	29
Turnover intention	16.60	6.30	7	36	29
IAT	−0.33	0.24	−1.04	0.31	1.35
SC-IAT	0.25	0.36	−0.82	1.1	1.88
					

### Correlation Analysis

As shown in Table 4, the correlation between implicit and explicit attitude scores can be used to determine whether overall attitudes are consistent. Explicit and implicit attitudes toward organizational loyalty did not match, although this correlation was not significant. This suggests that organizational loyalty is not consistent, and many employees want to leave their jobs, or that their work performance is poor. This could potentially have negative consequences for the organization.

**TABLE 4:** Correlation of total.

	Organizational loyalty	Organizational engagement	Turnover intention	IAT	SC-IAT
Organizational loyalty					
Organizational engagement	0.78*				
Turnover intention	−0.70*	−0.68*			
IAT	0.02	0.13	−0.05		
SC-IAT	0.08	0.08	−0.06	−0.12	

*p < 0.01.

### Analysis of Rank (Class)

Next, we sought to understand the effect of rank (class) on organizational attitude by assessing whether explicit attitudes correlate significantly with implicit attitudes. Among staff, the organizational engagement of new employees, as shown in Table 5-1, was significantly correlated with general IAT results. This was consistent with their implicit and explicit attitudes toward organizational engagement. These results imply that staff members have similar attitudes toward the organization. Therefore, staff will clearly recognize what they think and feel about the organization and will be motivated to stably adapt.

**TABLE 5-1:** Correlation of staff.

	Organizational loyalty	Organizational engagement	Turnover intention	IAT	SC-IAT
Organizational loyalty					
Organizational engagement	0.71**				
Turnover intention	−0.65**	−0.58**			
IAT	0.18	0.33**	–0.05		
SC-IAT	–0.04	0.08	–0.06	–0.08	

*p < 0.05, **p < 0.01.

**TABLE 5-2:** Correlation of assistant manager.

	Organizational loyalty	Organizational engagement	Turnover intention	IAT	SC-IAT
Organizational loyalty					
Organizational engagement	0.57*				
Turnover intention	−0.55*	−0.59**			
IAT	–0.11	0.05	–0.18		
SC-IAT	–0.09	–0.28	–0.06	–0.26	

* *p* < 0.05, ** *p* < 0.01.

**TABLE 5-3:** Correlation of manager.

	Organizational loyalty	Organizational engagement	Turnover intention	IAT	SC−IAT
Organizational loyalty					
Organizational engagement	0.88*				
Turnover intention	−0.72*	−0.76**			
IAT	–0.23	–0.18	–0.07		
SC-IAT	0.34*	0.42*	–0.06	–0.08	

*p < 0.05, **p < 0.01.

**TABLE 5-4:** Coincidence of rank (class).

	Organizational loyalty	Organizational engagement
Staff	X (Disagreement)	O (Agreement)
Assistant Manager	X (Disagreement)	X (Disagreement)
Manager	O (Agreement)	O (Agreement)

We found no significant correlation among all variables when we analyzed the responses of employees in the assistant manager class, as shown in Table 5-2. Because we observed disagreement between implicit and explicit attitudes within this class, their responses were likely to have been falsified or distorted. Their preferences and positive attitudes toward the organization were also significantly reduced relative to the other classes. In the case of the deputy general manager class, as shown in Table 5-3, both Organizational loyalty and Organizational engagement were significantly correlated with SC-IAT scores. This implies that their implicit and explicit attitudes are consistent with organizational totality and organizational engagement, that managers have stable and clear attitudes, and that this is consistent with the most favorable and active attitudes of all employees of the organization. When all results were combined, as shown in Table 5-4, the general staff members were shown to have a consistent attitude toward organizational engagement, and the managers’ scores revealed a consistent attitude toward organizational loyalty and organizational engagement. On the other hand, the assistant managers’ attitudes were inconsistent and their reactions unreliable, such that attitudes about commitment and loyalty to the organization were not apparent based on their responses.

When scores on multiple measures were correlated with measures of implicit attitude, we found that significant positive correlation with IAT scores and significant positive correlation with SC-IAT scores did not coincide. In particular, the responses from members of low ranks such as Staff showed significant static correlation with IAT outcomes, and responses of high ranks such as manager revealed significant static correlations with SC-IAT scores, underscoring the difference between the relativity and absoluteness of preference and approach. Due to the relatively short time for which the staff had been with the company, their judgments and thoughts are necessarily based on comparisons with their experiences in other companies. Therefore, their responses on explicit organizational loyalty measures revealed significant positive correlations with their IAT scores, which indicate relative preference and approach. On the other hand, managers are able to judge by absolute standards because their longer tenure with the company has enabled them to internalize their thoughts and confidence in their company more fully. Therefore, their scores show significant positive correlations with the absolute preference and approach measures of the SC-IAT.

## Conclusion

The research investigated organizational loyalty, which is generally difficult to operationally measure since employees may distort or falsify their self-report due to concerns about recrimination from their organization based on their answers. This study has yielded several meaningful findings. We first successfully developed an IAT-based tool, the OL-IAT (Organizational Loyalty-IAT), which specifically measures implicit attitudes toward organizational loyalty. This tool was developed according to methods employed in previous studies and was tailored to a specific target group. Since it measures implicit attitudes, which are unconscious reactions, it is difficult for respondents to distort their answers, so the results are highly reliable. Therefore, it is expected to be a useful measure for future organizational loyalty research. Second, when we investigated whether implicit and explicit attitudes toward organizational loyalty matched, we found no significant correlation, and the discrepancy in attitudes was apparent based on the nature of the responses. This once again confirms that self-reported questionnaires are susceptible to distortion by false responses when organizational loyalty is measured overtly (Gilbert and Ricketts, 2008). Therefore, the method of measuring implicit attitudes we have applied here can be used to enrich the accuracy of participants’ responses and capture their true attitudes more thoroughly. Third, when we examined differences in attitudes according to employee rank (class) in the organization, explicit and implicit attitudes toward organizational loyalty only matched each other within the manager class. Organizational engagement scores were consistent with each other within the manager and staff classes. On the other hand, all attitudes measured at the level of assistant managers did not agree, suggesting that the attitudes of assistant managers were ambiguous and unstable. Based on these findings, we concluded that managers had the most overtly consistent attitudes toward organizational loyalty, and so could be predicted to lead the most stable organizational life. Because the staff also displayed stable attitudes, we concluded that new employees are more likely to place a high degree of focus on the organization and demonstrate greater commitment to it. The assistant managers, on the other hand, had unstable attitudes, likely leading to a disordered organizational life because they evince less affection and passion for the organization than employees in other occupational classes.

Staff responses on organizational loyalty measures were inconclusive, but their engagement with the organization was well-represented by the OL-IAT. This may be due to the sense of accomplishment and confidence that they feel as new employees just beginning their careers. Therefore, it would be in the best interests of both the organization and the staff for the organization to invest in supporting new staff and providing them with incentives to foster their preference for the organization. Along with progression through the career path, the assistant manager phase is generally called “the most unstable period.” The expectation that assistant managers should assume a proxy role increases their burden of work and their boredom within the organization, both of which tend to discourage organizational loyalty or engagement. Therefore, organizations should aim to revive the assistant managers’ interest and sense of purpose within the corporate community and to alleviate their sense of helplessness and loss of will. Since the manager class occupies a very stable position within the organization, the attitudes of managers regarding loyalty and engagement tend to be clear and consistent. However, excessive organizational loyalty can weaken the unique sense of self, and thus their retirement may carry with it a high sense of personal loss. Bearing this in mind, the organization should provide personal career development programs to help them establish more independent personal identities.

There are limitations of this study, which must be considered in future studies. First, the small sample size makes it difficult to generalize these results to other groups. Increasing the number of participants and diversifying into groups that are more varied in their distribution and representation of different genders, departments, and industry types will increase generalizability in future work. Second, the sample is biased because it is targeted at specific company employees. Further, the proportion of male employees recruited from “P company” was very high, and while this adequately reflects the overall demographics of the steel industry, this imbalance likely obscures the influence of gender on organizational culture. It will also be necessary for the future to investigate many different companies within the same industry or to consider how organizational culture may differ in other business types, such as the oil industry. Third, it is necessary to expand the range of tool utilization. The comparison of unconscious measurements could be enhanced by incorporating such tactics as observer evaluation or physiological measurements. These strategies could yield more versatile and meaningful insights than simply comparing implicit association measures with explicit attitudes assessed through self-reported questionnaires. In future studies, the use of various measures alongside the IAT will enhance reliability and validity. Lastly, to improve the predictive validity of the OL-IAT, future studies should address the effect of implicit organizational loyalty attitudes on organizational satisfaction, organizational performance, and job performance (Otto and Mamatoglu, 2015; Alshraideh et al., 2017).

In this study, an effective method of measuring organizational loyalty was devised that could potentially be used to accelerate interventions to increase organizational loyalty. Organizations that foster high organizational loyalty among their employees are much more likely to secure excellent, stable talents, which in turn leads to employee growth and contributes to organizational development and performance. Greater attention and informed approaches to assessing organizational loyalty will positively affect the long-term growth of an organization.

## Data Availability Statement

The raw data supporting the conclusions of this article will be made available by the authors, without undue reservation.

## Ethics Statement

The studies involving human participants were reviewed and approved by Chung-Ang University. The patients/participants provided their written informed consent to participate in this study.

## Author Contributions

All authors listed have made a substantial, direct and intellectual contribution to the work, and approved it for publication.

## Conflict of Interest

The authors declare that the research was conducted in the absence of any commercial or financial relationships that could be construed as a potential conflict of interest.
